# Association Study of a Functional Variant on *ABCG2* Gene with Sunitinib-Induced Severe Adverse Drug Reaction

**DOI:** 10.1371/journal.pone.0148177

**Published:** 2016-02-25

**Authors:** Siew-Kee Low, Koya Fukunaga, Atsushi Takahashi, Koichi Matsuda, Fumiya Hongo, Hiroyuki Nakanishi, Hiroshi Kitamura, Takamitsu Inoue, Yoichiro Kato, Yoshihiko Tomita, Satoshi Fukasawa, Tomoaki Tanaka, Kazuo Nishimura, Hirotsugu Uemura, Isao Hara, Masato Fujisawa, Hideyasu Matsuyama, Katsuyoshi Hashine, Katsunori Tatsugami, Hideki Enokida, Michiaki Kubo, Tsuneharu Miki, Taisei Mushiroda

**Affiliations:** 1 Core for Genomic Medicine, RIKEN Center for Integrative Medical Sciences, Yokohama, Japan; 2 Faculty of Pharmacy, The University of Sydney, Sydney, NSW, Australia; 3 Institute of Medical Science, The University of Tokyo, Tokyo, Japan; 4 Kyoto Prefectural University of Medicine, Kyoto, Japan; 5 Sapporo Medical University, Sapporo, Japan; 6 Akita University School of Medicine, Akita, Japan; 7 Iwate Medical University, Morioka, Japan; 8 Yamagata University Faculty of Medicine, Yamagata, Japan; 9 Chiba Cancer Center, Chiba, Japan; 10 Osaka City University Graduate School of Medicine, Osaka, Japan; 11 Osaka Medical Center for Cancer and Cardiovascular Diseases, Osaka, Japan; 12 Kinki University Faculty of Medicine, Osakasayama, Japan; 13 Wakayama Medical University, Wakayama, Japan; 14 Kobe University Graduate School of Medicine, Kobe, Japan; 15 Yamaguchi University Graduate School of Medicine, Ube, Japan; 16 Shikoku Cancer Center, Matsuyama, Japan; 17 Graduate School of Medical Sciences, Kyushu University, Fukuoka, Japan; 18 Graduate School of Medical and Dental Sciences, Kagoshima University, Kagoshima, Japan; University de Minho, PORTUGAL

## Abstract

Sunitinib is a tyrosine kinase inhibitor and used as the first-line treatment for advanced renal cell carcinoma (RCC). Nevertheless, inter-individual variability of drug’s toxicity was often observed among patients who received sunitinib treatment. This study is to investigate the association of a functional germline variant on *ABCG2* that affects the pharmacokinetics of sunitinib with sunitinib-induced toxicity of RCC patients in the Japanese population. A total of 219 RCC patients were recruited to this pharmacogenetic study. *ABCG2* 421C>A (Q141K) was genotyped by using PCR-Invader assay. The associations of both clinical and genetic variables were evaluated with logistic regression analysis and subsequently receiver operating characteristic (ROC) curve was plotted. About 43% (92/216) of RCC patients that received sunitinib treatment developed severe grade 3 or grade 4 thrombocytopenia according to the National Cancer Institute-Common Terminology Criteria for Adverse Events version 3.0, the most common sunitinib-induced adverse reaction in this study. In the univariate analysis, both age (*P* = 7.77x10^-3^, odds ratio (OR) = 1.04, 95%CI = 1.01–1.07) and *ABCG2* 421C>A (*P* = 1.87x10^-2^, OR = 1.71, 95%CI = 1.09–2.68) showed association with sunitinib-induced severe thrombocytopenia. Multivariate analysis indicated that the variant *ABCG2* 421C>A is suggestively associated with severe thrombocytopenia (*P* = 8.41x10^-3^, OR = 1.86, 95% CI = 1.17–2.94) after adjustment of age as a confounding factor. The area under curve (AUC) of the risk prediction model that utilized age and *ABCG2* 421C>A was 0.648 with sensitivity of 0.859 and specificity of 0.415. Severe thrombocytopenia is the most common adverse reaction of sunitinib treatment in Japanese RCC patients. *ABCG2* 421C>A could explain part of the inter-individual variability of sunitinib-induced severe thrombocytopenia.

## Introduction

Molecular targeting drugs are the new generation of cancer chemotherapeutic agents that were used to interfere with protein that plays a critical role in tumor growth or progression. It is known that activation of tyrosine kinase increases tumor cell growth and proliferation, induces anti-apoptotic effects, as well as promotes angiogenesis and metastasis.[1] Additionally, activation of growth factors and protein kinase by somatic mutation in cancer cells is a common phenomenon in tumorigenesis.[2–4] Taken all these factors into consideration, inhibition of tyrosine kinase has become one of the major targets to develop cancer therapy.

Sunitinib (sunitinib malate; Sutent; Pfizer Inc, New York, NY) is an orally multitargeted tyrosine kinase inhibitor known to inhibit vascular endothelial growth factor receptors (VEGFRs), platelet-derived growth factor receptors (PDGFR), c-KIT, Fms-like tyrosine kinase 3 receptor (FLT3) and receptor encoded by the ret proto-oncogene.[5–7] Currently, sunitinib is given as first-line treatment to advanced renal cell carcinoma (RCC) and imatinib-resistant gastrointestinal stromal tumor (GIST). Although RCC patients revealed significant prolonged progression free survival and overall survival after administering sunitinib compared to interferon-alpha treatment in some randomized clinical trials,[8, 9] several common sunitinib-induced adverse events such as thrombocytopenia, hypertension, hand-foot syndrome, leucopenia and neutropenia were frequently observed.[8, 10–13]

There are inter-individual variability responses among patients who received sunitinib treatment, especially among the Asian patients. For instance, a study from Japan indicated more than 50% of Japanese RCC patients who received sunitinib developed severe thrombocytopenia (grade 3/4) compared to only less than 5% patients from a phase 3 trial reported from the United States of America.[8, 14] In addition, recent reports indicated approximately 80% of Japanese and Korean patients who received sunitinib treatment were forced to discontinue or reduce the dose during the therapy owing to the development of adverse events.[12, 15] This has raised the importance of identifying markers that could be used to predict individuals who are at risk in developing sunitinib-induced adverse drug reaction.

It is currently widely known that genetic variations on drug metabolism and pharmacokinetics-related protein attributed to the differences of efficacy and toxicity of specific drugs among different population. Previous studies have reported the associations of genetic variations in *VEGF*, *VEGFR1*, *VEGFR2*, *VEGFR3*, *ABCB1*, *NR1/2*, *NR1/3* and *CYP3A5* genes with sunitinib treatment outcome.[16–20] Notably, genetic polymorphisms on *VEGF*, *VEGFR*, *VEGFA* haplotype and *eNOS* shown to be associated with sunitinib-induced hypertension as well as variants on *ABCG2* associated with the risk of sunitinib related toxicity in mRCC patients.[16, 21, 22]

Breast cancer resistance protein (BCRP/ABCG2) is a transporter expressed in the small intestine and blood-brain barriers that mediates the efflux and regulates the pharmacokinetics of various drugs including tyrosine kinase inhibitors, particularly sunitinib.[23–25] A well-studied functional variant on *ABCG2*, 421C>A (Q141K, rs2231142) is known to result in significant reduction of transport activity, increased drug accumulation and consequent reduction in drug resistance due to the decreased efflux velocity of drug when comparing with ABCG2-transfected cells carrying the variant A allele to the wild-type C allele.[26] Because this variant is located within the ATP-binding cassette domain that regulates the ATP binding activity of ABCG2 protein, reduction of ATPase activity was observed in cells transfected with Q141K compared to the wild-type ABCG2.[27] Additionally, a recent study by Mizuno and colleagues has reported that this functional variant markedly affected the blood concentration of sunitinib and subsequently increased the systemic exposure of sunitinib that will cause the development of adverse events.[28] The aim of the current study is to investigate the association of *ABCG2* 421C>A with sunitinib-induced adverse events of RCC patients in the Japanese population.

## Material and Methods

### Sample populations

A total of 219 RCC patients were recruited from 15 Japanese medical institutes to participate in this study. We collected clinical information including age, gender, Eastern Cooperative Oncology Group (ECOG) performance status, treatment efficacy and the occurrence of various type of adverse events. The development of sunitinib-related adverse events was evaluated for six weeks (four-weeks-on and two-weeks-off periods). The grade of toxicity was classified in accordance with the National Cancer Institute-Common Terminology Criteria for Adverse Events (CTCAE) version 3.0. All the patients who enrolled in this study provided written informed consent in advance. This study was approved by the ethical committees from Kyoto Prefectural University of Medicine (Kyoto, Japan), Sapporo Medical University (Sapporo, Japan), Akita University School of Medicine (Akita, Japan), Iwate Medical University (Morioka, Japan), Yamagata University Faculty of Medicine (Yamagata, Japan), Chiba Cancer Center, (Chiba, Japan), Osaka City University Graduate School of Medicine (Osaka, Japan), Osaka Medical Center for Cancer and Cardiovascular Diseases (Osaka, Japan), Kinki University Faculty of Medicine (Osakasayama, Japan), Wakayama Medical University (Wakayama, Japan), Kobe University Graduate School of Medicine (Kobe, Japan), Yamaguchi University Graduate School of Medicine (Ube, Japan), Shikoku Cancer Center (Matsuyama, Japan), Graduate School of Medical Sciences, Kyushu University (Fukuoka, Japan), Graduate School of Medical and Dental Sciences, Kagoshima University (Kagoshima, Japan) that are involved in samples collection and RIKEN Center for Integrative Medical Sciences (Yokohama, Japan) as well as Institute of Medical Science, The University of Tokyo (Tokyo, Japan) that carried out the genetic study.

### Genotyping of *ABCG2* functional SNP

To obtain the genotype of *ABCG2* 421C>A (Q141K), PCR amplification was firstly carried out with specific primers (Forward primer-ACTGCAGGTTCATCATTAGC; Reverse primer-TTCCACATTACCTTGGAGTCTG) flanking *ABCG2* 421C>A under the condition of initial denaturation at 95°C for 2 min, followed by 40 cycles at 95°C for 15 sec, 60°C for 45 sec and 72°C for 1.5 min using GeneAmp 9700 (Applied Biosystems, Foster City, CA). After PCR amplification, the product was diluted 10-fold and used as templates for Invader assay. Invader assay was performed with ABI PRISM 7900 (Applied Biosystems) according to the protocol recommended by the Third Wave Technologies (Madison, WI).

### Statistical analysis

To evaluate the association of clinical variables with sunitinib-induced adverse events, we applied univariate analysis (Generalized linear model) to observe the association of age, gender, ECOG performance status and RCC histology with different adverse reaction. We applied logistic regression analysis to observe the association of *ABCG2* 421C>A with several adverse event phenotypes that include: the increase levels of ALT and AST, diarrhea, fever, hand-foot syndrome, hypertension, hypothyroidism, leucopenia, neutropenia and thrombocytopenia. Multivariate analyses were performed to evaluate the association of ABCG2 421C>A with adverse reaction after adjustment of associated clinical variables, which are gender for leucopenia, ECOG performance status for hand-foot syndrome and age for proteinuria as well as thrombocytopenia. We also evaluated the association of ABCG2 421C>A with adverse reaction after adjustment all the clinical variables.

To develop a risk prediction model, we scored each of the samples with score of 2 to individual who possess two risk alleles, 1 to that with one risk allele and 0 to that with no risk allele. Subsequently, we created the prediction model by utilizing unconditional logistic regression in which we multiplied the respective regression coefficient (weight) to the number of risk alleles of the SNP that each individual possess and to the associated clinical variables. In the current study, the joined effect of age and *ABCG2* 421C>A associated with severe thrombocytopenia was evaluated according to the formula as follow:

$$\log\left(OR\right)=\log\left(\frac{p(x)}{1-p(x)}\right)=\beta_{0}+\beta_{Age}X_{Age}+\beta_{SNP}X_{SNP}$$

$$\beta_{0}=Intercept=-3.32679$$

$$\beta_{Age}=\text{Regression coefficient for age}=0.04249$$

$$X_{Age}=\text{Age}$$

$$\beta_{SNP}=\text{Regression coefficient for SNP}=0.61829$$

$$X_{SNP}=ABCG\;2421C>\text{A genotypes}\;\left(0,1,2\right)$$

ROC curve was plotted with true positive rate (sensitivity) versus false positive rate (1-specificity) and area under curve (AUC) was used to evaluate how well the prediction model could distinguish between the two diagnostic groups (with or without adverse events). Positive predictive value (PPV) and negative predictive value (NPV) were calculated.

All the analysis was carried out using R statistical environment 3.0.1. We utilized R package Epi and pROC to estimate AUC and plot ROC curve, respectively.

## Results

We evaluated both clinical and genetic variables associated with various sunitinib-induced adverse events in 219 RCC patients. Clinical characteristics of study patients were summarized in Table 1. Among these patients, 43% (92/216) of patients developed grade 3 and 4 thrombocytopenia; 25% (52/211) developed grade 3 and 4 neutropenia; 20% (42/207) developed grade 3 and 4 hypertension; 17% (37/217) developed grade 3 and 4 leucopenia; 8.4% (18/215) has increased AST/ALT level and 6.5% (14/214) developed grade 3 hand-foot syndrome. Among various adverse reactions, severe thrombocytopenia is the most common adverse reaction in RCC patients receiving sunitinib treatment.

**Table 1:** Patient demographics and toxicity grades of this study.

Characteristic			
**Total**	219		
**Median age, years (range)**	63 (32–83)		
**Gender**		**RCC histology**	
Male	161	Clear cell carcinoma	176
Female	58	Papillary renal cell carcinoma	8
		Chromophobe cell carcinoma	1
**ECOG performance status**		Cystic renal cell carcinoma	1
0	159	Spindle cell carcinoma	5
1	45	Granular cell carcinoma	2
2	11	Others	13
3	2	Unknown	13
Missing	2		
**Type of toxicity**			
**Thrombocytopenia**		**Increase of AST (GOT)/ALT (GPT)**	
Grade 4	12	Grade 4	2
Grade 3	80	Grade 3	16
Grade 2	51	Grade 2	33
Grade 1	45	Grade 1	77
Grade 0	28	Grade 0	87
NA	3	NA	4
**Hypertension**		**Proteinuria**	
Grade 4	2	Grade 4	1
Grade 3	40	Grade 3	5
Grade 2	38	Grade 2	29
Grade 1	11	Grade 1	36
Grade 0	116	Grade 0	145
NA	12	NA	3
**Leucopenia**		**Hand-foot syndrome**	
Grade 4	1	Grade 3	14
Grade 3	36	Grade 2	56
Grade 2	88	Grade 1	49
Grade 1	40	Grade 0	95
Grade 0	52	NA	5
NA	2		
**Neutropenia**		**Hypothyroidism**	
Grade 4	2	Grade 3	2
Grade 3	50	Grade 2	79
Grade 2	54	Grade 1	30
Grade 1	14	Grade 0	98
Grade 0	91	NA	10
NA	8		
**Diarrhea**		**Fever**	
Grade 4	1	Grade 3	1
Grade 3	1	Grade 2	13
Grade 2	17	Grade 1	32
Grade 1	37	Grade 0	167
Grade 0	158	NA	6
NA	5		

Among the clinical variables, we observed that ECOG performance status associated with the occurrence of hand-foot syndrome (*P* = 4.87x10^-2^, odds ratio (OR) = 0.576, 95%CI = 0.332–0.997), gender (females) associated with increased risk for severe leucopenia (*P* = 1.46x10^-2^, OR = 2.50, 95%CI = 1.20–5.23) and age as one of the associated factors that affects the occurrence of proteinuria (*P* = 4.22x10^-2^, OR = 1.04, 95%CI = 1.00–1.083) and severe thrombocytocypenia (*P* = 7.77x10^-3^, OR = 1.04, 95%CI = 1.01–1.07) (Table 2).

**Table 2:** Association of clinical variables with sunitinib-induced adverse events.

Adverse event	Gender			ECOG performance status			Age			RCC histology		
	P-value	OR	95%CI	P-value	OR	95%CI	P-value	OR	95%CI	P-value	OR	95%CI
Increased AST/ALT	1.83E-01	1.975	0.725–5.380	9.82E-01	1.009	0.458–2.223	6.77E-01	0.990	0.946–1.037	3.62E-01	0.576	0.176–1.886
Diarrhea	2.71E-01	0.49	0.137–1.748	2.16E-01	0.494	0.162–1.510	7.25E-01	0.992	0.949–1.037	6.36E-01	1.445	0.315–6.635
Fever	2.73E-01	0.426	0.092–1.963	8.33E-01	1.096	0.468–2.568	1.55E-01	0.965	0.918–1.014	9.38E-01	1.063	0.226–5.010
Hand-foot syndrome	6.55E-01	1.157	0.610–2.192	4.87E-02	0.576	0.332–0.997	2.46E-01	0.984	0.957–1.011	9.01E-01	1.054	0.463–2.397
Hypertension	9.86E-01	1.007	0.466–2.176	4.06E-01	1.241	0.745–2.068	2.73E-01	1.019	0.985–1.055	8.02E-01	0.882	0.331–2.355
Hypothyroidism	5.02E-01	1.241	0.661–2.330	7.71E-01	0.931	0.574–1.509	8.86E-01	0.998	0.972–1.025	4.05E-01	0.717	0.328–1.568
Leucopenia	1.46E-02	2.504	1.199–5.228	6.50E-01	1.136	0.656–1.965	6.47E-02	1.036	0.998–1.076	7.92E-02	0.440	0.176–1.100
Neutropenia	2.81E-01	1.466	0.732–2.935	1.62E-01	0.656	0.363–1.185	5.28E-01	1.010	0.979–1.042	1.73E-01	0.554	0.236–1.296
Proteinuria	5.61E-01	0.776	0.331–1.823	6.54E-01	1.137	0.649–1.992	4.22E-02	1.041	1.001–1.083	7.53E-01	1.199	0.387–3.712
Thrombocytopenia	9.27E-01	1.029	0.560–1.890	2.09E-01	0.745	0.470–1.180	7.77E-03	1.039	1.010–1.069	1.91E-01	0.595	0.273–1.296

Analysis was examined with patients who developed adverse reaction of increased AST/ALT (grade 3 and 4 versus others), diarrhea (grade2 to 4 versus others), fever (grade2 to 4 versus others), hand-foot syndrome (grade2 to 4 versus others), hypertension (grade 3 and 4 versus others), hypothyroidism (grade2 to 4 versus others), leucopenia (grade 3 and 4 versus others), neutropenia (grade 3 and 4 versus others), proteinuria (grade2 to 4 versus others) and thrombocytopenia (grade 3 and 4 versus others). Abbreviations: OR, odds ratio; CI, confidence interval.

To evaluate the association of *ABCG2* 421C>A with various sunitinib-induced adverse drug reactions, we performed univariate logistic regression analysis and observed that *ABCG2* 421C>A is associated with severe thrombocytopenia (*P* = 1.87x10^-2^, OR = 1.71, 95% CI = 1.09–2.68), fever (*P* = 1.59x10^-2^, OR = 2.85, 95% CI = 1.22–6.66) and increased levels of AST and ALT (*P* = 4.21x10^-2^, OR = 2.18, 95% CI = 1.03–4.64). The association remained suggestively significant with severe thrombocytopenia (*P* = 5.18x10^-3^, OR = 2.26, 95% CI = 1.28–4.00), fever (*P* = 1.78x10^-2^, OR = 2.83, 95% CI = 1.20–6.70) and increased levels of AST and ALT (*P* = 3.71x10^-2^, OR = 3.40, 95% CI = 1.08–10.72) after adjusting the significant clinical variables (gender for leucopenia, ECOG performance status for hand-foot syndrome and age for proteinuria as well as thrombocytopenia) as confounding factors that might affect the association (Table 3). Although the association of *ABCG2* 421C>A is not statistically significant but it remained suggestively associated with severe thrombocytopenia in the Japanese population after Bonferroni correction (Threshold = 0.05/10 independent phenotypes = 0.005) as multiple testing. We also evaluated *ABCG2* 421C>A by incorporating age, gender, ECOG status and RCC histology as confounding factors as shown in S1 Table.

**Table 3:** Association of *ABCG2* 421C>A,Q141K (rs2231142) with various sunitinib-induced adverse events.

Adverse event	Case				Control				Allele_A_frequency		Univariate				Multivariates			
	AA	AC	CC	Total	AA	AC	CC	Total	Case	Control	P-value	OR	L95	U95	P-value	OR	L95	U95
Increased AST/ALT	2	12	4	18	13	87	97	197	0.444	0.287	4.21E-02	2.184	1.028	4.640	4.21E-02	2.184	1.028	4.64
Diarrhea	2	10	7	19	13	88	94	195	0.368	0.292	3.07E-01	1.468	0.703	3.067	3.07E-01	1.468	0.703	3.067
Fever	2	10	2	14	13	88	98	199	0.500	0.286	1.59E-02	2.845	1.216	6.657	1.59E-02	2.845	1.216	6.657
Hand-foot syndrome	6	35	29	70	9	65	70	144	0.336	0.288	2.90E-01	1.283	0.809	2.035	3.69E-01	1.24	0.776	1.98
Hypertension	3	22	17	42	11	75	79	165	0.333	0.294	4.44E-01	1.238	0.717	2.135	4.57E-01	1.23	0.713	2.124
Hypothyroidism	6	42	33	81	8	53	67	128	0.333	0.270	1.45E-01	1.402	0.891	2.208	1.45E-01	1.402	0.89	2.208
Leucopenia	1	16	20	37	14	84	82	180	0.243	0.311	2.24E-01	0.687	0.375	1.258	2.78E-01	0.712	0.385	1.316
Neutropenia	3	23	26	52	12	74	73	159	0.279	0.308	5.53E-01	0.856	0.512	1.431	5.53E-01	0.856	0.512	1.431
Proteinuria	3	18	14	35	11	82	88	181	0.343	0.287	3.25E-01	1.339	0.749	2.396	2.13E-01	1.456	0.806	2.631
Thrombocytopenia	8	50	34	92	7	50	67	124	0.359	0.258	1.87E-02	1.710	1.093	2.675	8.41E-03	1.856	1.172	2.939

Association was examined by logistic regression analysis after adjustment of associated clinical variables (gender or ECOG performance status, age or RCC histology) identified from univariate analysis. Analysis was performed with patients who developed adverse reaction of increased AST ALT (grade 3 and 4 versus others), diarrhea (grade2 to 4 versus others), fever (grade2 to 4 versus others), hand-foot syndrome (grade2 to 4 versus others), hypertension (grade 3 and 4 versus others), hypothyroidism (grade2 to 4 versus others), leucopenia (grade 3 and 4 versus others), neutropenia (grade 3 and 4 versus others), proteinuria (grade2 to 4 versus others) and thrombocytopenia (grade 3 and 4 versus others). Abbreviations: OR, odds ratio; L95, lower bound of a 95% confidence interval; U95, upper bound of a 95% confidence interval.

**Fig 1:**
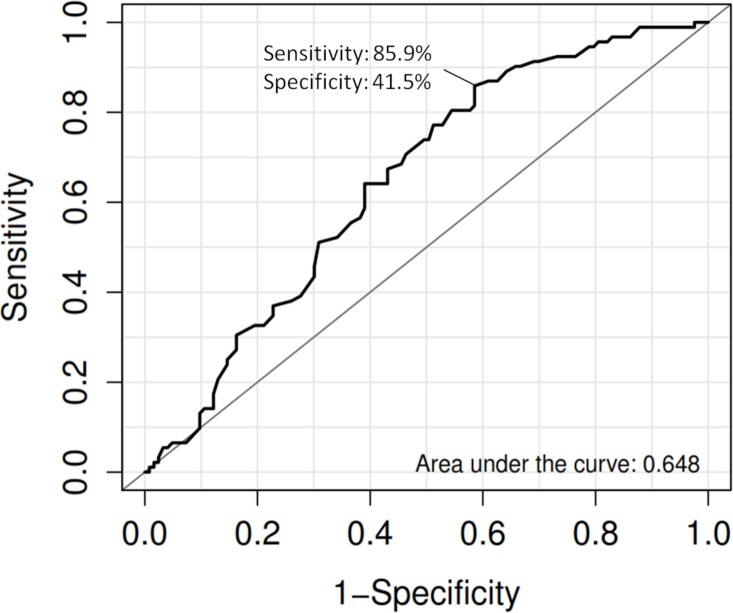
ROC curve of the combined effects of *ABCG2* 421C>A (Q141K) and age with severe thrombocytopenia.

**Fig 2:**
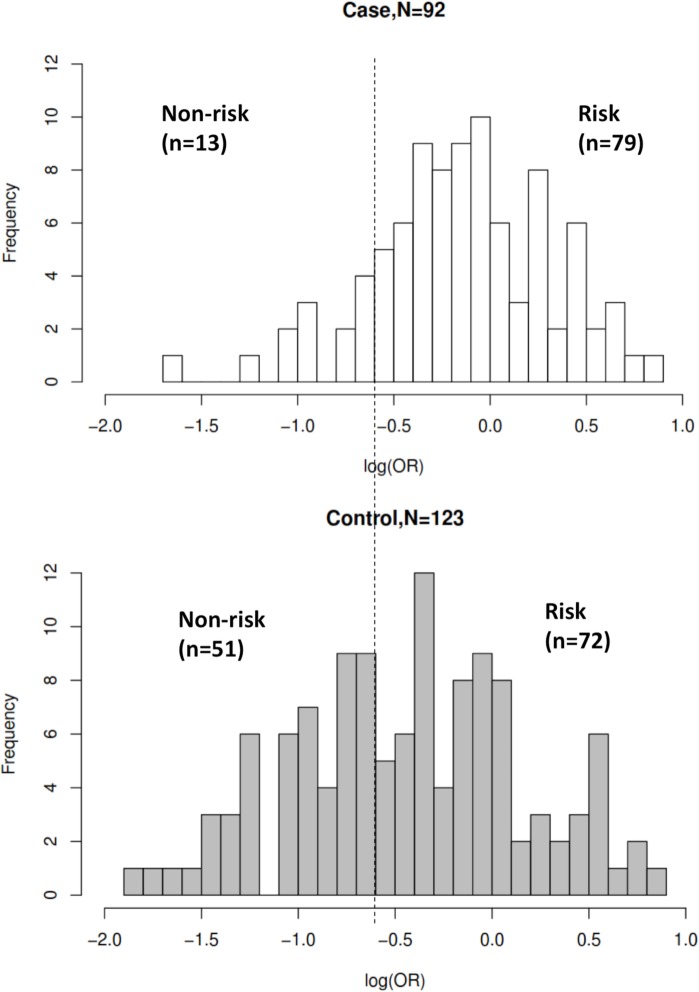
Histogram plot with case-control frequency versus distribution of log (OR). Threshold of the plot was obtained from AUC curve with optimal sensitivity and specificity. Significant difference (*P* = 1.12x10^-5^) was observed between risk and non-risk of case-control in this study with odds ratio of 4.30 (95%CI = 2.07–9.10).

The AUC of the risk prediction model by utilizing age and *ABCG2* 421C>A with sunitinib-induced severe thrombocytopenia was 0.648 with sensitivity of 0.859 and specificity of 0.415 (Fig 1). Fig 2 showed the frequency distribution of cases and controls against log(OR) value. When threshold was set with the optimal sensitivity and specificity obtained from AUC, the OR between case-control was 4.30 (95%CI = 2.07–9.10). The PPV and NPV of the model are 0.434 and 0.849, respectively, after incorporating the prevalence of thrombocytopenia as 34.35% that was reported in the post-marketing surveillance study in Japanese patients by Pfizer Inc (http://www.sutent.jp/).

## Discussion

The current study is the first study to evaluate both clinical and genetic variable, *ABCG2* 421C>A, that were associated with sunitinib-induced thrombocytopenia in the Japanese population. Our study suggested that age and *ABCG2* 421C>A (Q141K) functional variant are significantly associated with sunitinib-induced severe thrombocytopenia. By utilizing the current estimated prediction model with both clinical variable (age) and genetic variable (*ABCG2* 421C>A), it might become possible to lower the incidence of severe thrombocytopenia (grade 3 and above) induced by sunitinib from 34.35% to 4.84% by excluding the patients judged to be the risk type from the sunitinib treatment.

A limitation of this study is that we did not take into the account of administered sunitinib dosage (preferably cumulative dosage) that affected the occurrence of adverse drug reactions as the collection of such information was principally challenging for a multicenter study to obtain a uniform clinical phenotype across different centers.

*ABCG2* 421C>A leads to the replacement of glutamine (polar-neutral) with lysine (positively charged) within ATP-binding domain, which is one of the most important domains that regulates ATP binding that supplies the energy to pump substrates against a concentration gradient. This phenomenon was supported by Mizuarai S et al., which indicated the reduction of transporter’s efflux activity that was indirectly measured by ATPase activity with cells transfected with ABCG2 Q141K variants compared with the wild-type.[27] Importantly, compared to wild-type cells, ABCG2 Q141K variant cells presented markedly lower expression of ABCG2, which might contribute to the increased toxicity to anticancer drugs.[26] This variant is also known to affect the pharmacokinetics of certain drugs, such as gefitinib, irinotecan and sulfasalazine, and subsequently increased these drug-induced toxicity, which further suggest the significance of this variant for therapeutic implications.[29]

The identification of *ABCG2* 421C>A associated with severe thrombocytopenia is particularly of importance as the Asian population possess relatively high frequency of this variant, variant allele A frequency is 0.311 and 0.289 in Japanese and Han Chinese populations, respectively, as compared to other non-Asian populations, allele A frequency is only 0.117 in Caucasian population and non-polymorphic in African population according to the SNP database from NCBI (http://www.ncbi.nlm.nih.gov/SNP/snp_ref.cgi?rs=2231142). Importantly, post marketing surveillance report from Pfizer showed that the incidence of sunitinib-induced severe (G3 and/or G4) thrombocytopenia is relatively lower (9%) in the European population as compared to the Japanese population (34%), which further fortify the suggestion of this variant to explain partly the inter-individual variability in the response to sunitinib treatment among different ethnic groups (http://www.sutent.jp and http://www.accessdata.fda.gov/drugsatfda_docs/label/2013/021938s024s025lbl.pdf). The finding of this study is in agreement with a recent study reported from Korean population with 65 RCC patients who received sunitinib therapy, which also reported a significant association of *ABCG2* 421C>A with grade 3 and grade 4 thrombocytopenia (*P-*value = 0.04, OR = 9.90).[22]

The AUC value utilizing the two parameters (age and *ABCG2* 421C>A variant) is 0.648, which indicates that the current prediction model required further improvement by identifying additional clinical and genetic factors associated with sunitinib-induced severe thrombocytopenia. Nevertheless, this study has demonstrated the contribution of both clinical and genetic variants that are useful and important to therapeutic implication of sunitinib treatment. Further investigation and validation of this finding are essential as the ultimate endpoint of this study is to identify patients who required dosage reduction or selection of alternative treatment as well as to predict the occurrence of adverse event that clinical professional could prepare for termination of treatment due to toxicity.

## Supporting Information

S1 Table Association of ABCG2 421C>A,Q141K (rs2231142) with various sunitinib-induced adverse events after adjusting age, gender, ECOG performance and RCC histology. (PDF)
