# Supplementary material for: Association Study of a Functional Variant on ABCG2 Gene with Sunitinib-Induced Severe Adverse Drug Reaction
Source: PLoS One. 2016 Feb 25;11(2):e0148177. doi: 10.1371/journal.pone.0148177 (PMC4767438; doi:10.1371/journal.pone.0148177)
Supplement: S1 Table — (PDF) [file pone.0148177.s001.pdf]

S1 Table: Association of ABCG2 421C>A,Q141K (rs2231142) with various sunitinib-induced adverse events after adjusting age, gender, ECOG performance and RCC histology

Adverse event	Multivariates			
	P-value	OR	L95	U95
Increased AST/ALT	3.60E-02	2.307	1.056	5.039
Diarrhea	2.80E-01	1.543	0.702	3.389
Fever	1.98E-02	2.833	1.180	6.800
Hand-foot syndrome	3.74E-01	1.247	0.767	2.026
Hypertension	3.55E-01	1.314	0.737	2.344
Hypothyroidism	1.66E-01	1.400	0.870	2.252
Leucopenia	4.88E-01	0.788	0.403	1.543
Neutropenia	4.21E-01	0.798	0.460	1.383
Proteinuria	4.11E-01	1.299	0.696	2.423
Thrombocytopenia	1.02E-02	1.886	1.162	3.060

Association was examined by logistic regression analysis, multivariate analysis were performed after adjustment of clinical variables (age, gender, ECOG performance status and RCC histology). Analysis was performed with patients who developed adverse reaction of increased AST ALT (grade 3 and 4 versus others), diarrhea (grade2 to 4 versus others), fever (grade2 to 4 versus others), hand-foot syndrome (grade2 to 4 versus others), hypertension (grade 3 and 4 versus others), hypothyroidism (grade2 to 4 versus others), leucopenia (grade 3 and 4 versus others), neutropenia (grade 3 and 4 versus others), proteinuria (grade2 to 4 versus others) and thrombocytopenia (grade 3 and 4 versus others). Abbreviations: OR, odds ratio; L95, lower bound of a 95% confidence interval; U95, upper bound of a 95% confidence interval.
